# Sensory Neurons of the Dorsal Root Ganglia Become Hyperexcitable in a T-Cell-Mediated MOG-EAE Model of Multiple Sclerosis

**DOI:** 10.1523/ENEURO.0024-19.2019

**Published:** 2019-04-01

**Authors:** Muhammad Saad Yousuf, Myung-chul Noh, Timothy N. Friedman, Kasia Zubkow, John Christy Johnson, Gustavo Tenorio, Harley T. Kurata, Peter A. Smith, Bradley J. Kerr

**Affiliations:** 1 Neuroscience and Mental Health Institute, University of Alberta, Edmonton, Alberta T6G 2E1, Canada; 2 Department of Pharmacology, University of Alberta, Edmonton, Alberta T6E 2H7, Canada; 3 Department of Anesthesiology and Pain Medicine, University of Alberta, Edmonton, Alberta T6G 2G3, Canada

**Keywords:** DRG, EAE, electrophysiology, MS, pain

## Abstract

Multiple sclerosis (MS) is an autoimmune, demyelinating disease of the central nervous system. Patients with MS typically present with visual, motor, and sensory deficits. However, an additional complication of MS in large subset of patients is neuropathic pain. To study the underlying immune-mediated pathophysiology of pain in MS we employed the myelin oligodendrocyte glycoprotein (MOG)-induced experimental autoimmune encephalitis (EAE) model in mice. Since sensory neurons are crucial for nociceptive transduction, we investigated the effect of this disease on sensory neurons of the lumbar dorsal root ganglia (DRG). Here, we report the disease was associated with activation of the complement system and the NLRP3 inflammasome in the DRG. We further observe a transient increase in the number of complement component 5a receptor 1-positive (C5aR1+) immune cells, CD4+ T-cells, and Iba1+ macrophages in the DRG. The absence of any significant change in the levels of mRNA for myelin proteins in the DRG and the sciatic nerve suggests that demyelination in the PNS is not a trigger for the immune response in the DRG. However, we did observe an induction of activating transcription factor 3 (ATF3) at disease onset and chronic disruption of cytoskeletal proteins in the DRG demonstrating neuronal injury in the PNS in response to the disease. Electrophysiological analysis revealed the emergence of hyperexcitability in medium-to-large (≥26 µm) diameter neurons, especially at the onset of MOG-EAE signs. These results provide conclusive evidence of immune activation, neuronal injury, and peripheral sensitization in MOG-EAE, a model classically considered to be centrally mediated.

## Significance Statement

The pathogenesis of multiple sclerosis (MS) involves central nervous system inflammation, demyelinating plaques, and neurodegeneration. Neuropathic pain is a common symptom of MS thought to occur as a result of disease pathology in the central nervous system. On the contrary, here, we demonstrate that the PNS in myelin oligodendrocyte glycoprotein (MOG)-induced experimental autoimmune encephalomyelitis (EAE), a currently used T-cell mediated model of MS, experiences transient inflammation, cellular injury, and chronic cytoskeletal disruption. Furthermore, putative myelinating, medium-to-large diameter sensory neurons of the dorsal root ganglia (DRG) show aberrant electrophysiological properties. Our data suggests that pain in MOG-EAE may at least in part be due to disease-mediated sensitization of primary afferents, ultimately enhancing sensory input into the spinal cord.

## Introduction

Multiple sclerosis (MS) is an autoimmune disorder that is characterized by inflammation and demyelinating lesions that target the brain and spinal cord (SC), ultimately leading to neurodegeneration (Hemmer et al., 2015). Common symptoms of MS involve visual, motor, and sensory changes including neuropathic pain (Benson and Kerr, 2014). Over half of MS patients will experience neuropathic pain at some point during the course of their disease (Drulovic et al., 2015). Neuropathic pain is associated with disability, depression, and anxiety which ultimately contribute to a poorer quality of life (Drulovic et al., 2015). Current therapies to alleviate pain in this population have largely been ineffective (Truini et al., 2013).

While the pathologic features of MS and experimental autoimmune encephalomyelitis (EAE) are focused in the CNS, sensory neurons residing in the dorsal root ganglia (DRG) and trigeminal ganglia (TG) also undergo significant changes in response to chronic, concomitant CNS inflammation (Duffy et al., 2016; Thorburn et al., 2016; Yousuf et al., 2017). A significant body of literature emerging from the rodent model of MS, myelin oligodendrocyte glycoprotein (MOG)-induced EAE, demonstrates that the DRG and TG undergo major pathologic changes with disease progression (Duffy et al., 2016; Thorburn et al., 2016; Yousuf et al., 2017). Indeed, there is evidence of leukocyte invasion and increased pro-inflammatory cytokine expression in the sensory ganglia of rats and mice with EAE (Melanson et al., 2009; Begum et al., 2013; Duffy et al., 2016; Frezel et al., 2016; Rodrigues et al., 2016). It is unclear, however, how the physiologic properties of the primary sensory neurons of the DRG are affected in the diseased state.

Neuropathic pain is postulated to involve sensitization, or enhanced signaling, along the primary afferent pain pathway. Nociceptors are a specialized class of neural cell in the PNS that encode painful stimuli across various modalities such as noxious heat, chemicals, and mechanical stimulation. In concert with other sensory neurons, nociceptors inform the central nervous system about the nature, location, and intensity of the painful stimulus. Primary afferents are classified across various combinations of myelination status, response characteristics, cell soma size, and specific molecular markers (Basbaum et al., 2009). Among these, nociceptors are generally classified as unmyelinated, small-diameter C-fibers or lightly myelinated, medium-diameter A-δ fibers that respond to multiple modalities to produce slow pain (C-fibers) and fast pain (A-δ fibers; Davis et al., 1993; Slugg et al., 2000). In contrast, mechanoreceptors and proprioceptors are heavily myelinated, have a larger diameter, and respond to touch and position in space. Recent attempts to classify sensory neurons based on molecular signatures have revealed 11 different subsets (Usoskin et al., 2015). Enhancement of the response properties of sensory neurons, known as peripheral sensitization, due to inflammation or injury can often lead to hyperalgesia (increased sensitivity to painful stimulus) and allodynia (pain from a non-noxious stimulus; Abdulla and Smith, 2001a; Ma et al., 2003; Stemkowski et al., 2015; Moy et al., 2017).

In this study, we aimed to study the impact of the MOG-EAE disease on the primary sensory neurons of the lumbar DRG in mice. We find that at the onset of disease, DRGs become inflamed with immune cell invasion followed by cytoskeletal disruption at more chronic time points. Electrophysiological analysis reveals that medium-to-large diameter neurons become hyperexcitable. They exhibit increased action potential (AP) firing, reduced rheobase, and decreased cumulative spike latencies in the diseased condition. Altogether, this study demonstrates that there are significant functional alterations in the DRG in response to MOG-EAE contesting the commonly held notion that the pathology of MOG-induced EAE is limited to the CNS.

## Materials and Methods

### EAE induction and scoring

MOG-EAE was elicited by subcutaneously injecting 50 µg of MOG (MOG_35-55_; Peptide Synthesis Facility, Stanford University), emulsified in complete Freund’s adjuvant (CFA; 1.5 mg/ml) in the hind flank. Eight- to 10-week-old female C57BL/6 mice were used in this study (*n* = 90; Charles River). Mice were examined daily for clinical signs of the disease and classified using the following criteria: grade 0, no signs; grade 1, paralyzed tail; grade 2, mild hindlimb weakness; grade 3, severe hindlimb weakness; grade 4, complete hindlimb paralysis; grade 5, moribund. MOG-EAE mice were grouped according to their disease progression: EAE onset (at appearance of clinical symptoms, grade 1), and chronic (day 21 post-induction; average disease score: 2.67 ± 0.22). A set of CFA-only administered mice were used as control for EAE induction. Two intraperitoneal injections of pertussis toxin, *Bordatella pertussis* (List Biological Labs) were also administered to all mice on the day of induction and 48 h thereafter.

All animal experiments were performed according to the national Council on Animal Care’s Guidelines and Policies with approval from the institute’s Health Sciences Animal Care and Use Committee.

### Tissue harvesting and storage

Mice were euthanized by intraperitoneal pentobarbital overdose (Euthansol, 0.1 ml of 340 mg/ml) followed by intracardiac flush with 0.9% saline. For immunohistochemical (IHC) experiments, the cadaver was transcardially perfused with 4% paraformaldehyde (PFA) in 0.1 M phosphate buffer (PB). Otherwise, lumbar DRGs (L3-L6) were extracted from euthanized animals promptly and snap frozen in liquid nitrogen. Similarly, the lumbar SC was extracted and the dorsal (dSC) and ventral halves dissected using a scalpel blade.

### Polymerase chain reaction

Total RNA was extracted from tissue samples using Qiazol (QIAGEN, 79306) and RNeasy Lipid Tissue Mini kit (QIAGEN, 74804); 200 ng of total RNA was subjected to DNase I treatment (Invitrogen, 18068-015) followed by Superscript III reverse transcription (Invitrogen, 18080-044) using oligo-dT_12-18_ primers (Invitrogen, 18418-012). PCR reactions (20 µl) were performed using Fast SYBR Green MasterMix (Applied Biosystems, 4385612) on Bio-Rad CFX96 thermocycler with *Ppia* as a housekeeping gene. Primers have been summarized in Table 1.

**Table 1.** qRT-PCR primers used in this study

Gene ID	FWD/REV	Sequence (5’–3’)
*Ppia*	FWD	GAGCTGTTTGCAGACAAAGTTC
	REV	CCCTGGCACATGAATCCTGG
*C3*	FWD	GAGGCACATTGTCGGTGGTG
	REV	CCAGGATGGACATAGTGGCG
*C3ar1*	FWD	CTCAGCAACTCGTCCAATGC
	REV	CCATGGCTCAGTCAAGCACA
*C5ar1*	FWD	CTTCCTTCAGAAGAGTTGCCTG
	REV	AGCTGCTGTTATCTATGGGGTC
*Nlrp3*	FWD	ATTACCCGCCCGAGAAAGG
	REV	TCGCAGCAAAGATCCACACAG
*Casp1*	FWD	ACAAGGCACGGGACCTATG
	REV	TCCCAGTCAGTCCTGGAAATG
*Il1b*	FWD	GCAACTGTTCCTGAACTCAACT
	REV	ATCTTTTGGGGTCCGTCAACT
*Il18*	FWD	ACTTTGGCCGACTTCACTGT
	REV	GGGTTCACTGGCACTTTGAT
*Mbp*	N/A	QIAGEN, PPM04745F
*Pmp22*	N/A	QIAGEN, PPM05053F
*Mpz*	N/A	QIAGEN, PPM41824A
*Mog*	N/A	QIAGEN, PPM33328B

### Western blotting

Western blotting was performed as previously described (Yousuf et al., 2017) with slight modifications. Briefly, tissue samples were homogenized and diluted to 1 µg/µl in RIPA (25 mM Tris, 150 mM NaCl, 0.1% SDS, 0.5% Na deoxycholate, and 1% NP-40) with protease (cOmplete EDTA-free, Roche, 04693159001) and phosphatase inhibitors (PhosSTOP, Roche, 04906837001). Samples were diluted in 4× Laemmli buffer (Bio-Rad, 1610747) with dithiothreitol (50 mM final concentration, Bio-Rad, 1610610) and boiled for 10 min before loading 16 µg of sample onto 4–20% Mini-PROTEAN TGX precast gels (Bio-Rad, 4561093DC). Gels were run at 150 V for 60 min and transferred onto PVDF membranes with 300 mA over 60 min. Membranes were blocked in 5% BSA in PBS-Tween (0.5% Tween 20 in 1× PBS) followed by overnight incubation at 4°C with primary antibody dissolved in 1% BSA in PBS-Tween. Membranes were further washed with PBS-Tween (3×, 10 min per wash) and incubated with secondary antibody in 1% BSA in PBS-Tween for 1 h at room temperature. Membranes were then washed with PBS-Tween (3×, 10 min per wash) and visualized using electrochemiluminescence (ECL; GE, 45000875) with Bio-Rad ChemiDoc XRS+ system. Membranes were stained with Coomassie Brilliant Blue (Bio-Rad, 1610400) to obtain total protein levels as loading control. Antibodies are summarized in Table 2.

**Table 2.** Antibodies used in this study

Antibody	Host	Source	Dilution factor
CD4	Rt	Bio-Rad, MCA2691	1:200
CD88 (C5aR1)	Rt	Bio-Rad, MCA2456GA	1:500
IBA-1	Rb	Wako, 019-19741	1:500
p-NFH (IHC)	Ck	ThermoFisher, PA1-10002	1:5000
ATF3 (IHC)	Rb	Santa Cruz, SC-188	1:200
NFH (WB)	Ms	Covance, 14974402	1:1000
Tau	Rb	Abcam, ab64193	1:200
Kinesin	Ms	Millipore, MAB1614	1:500
α-Tubulin	Rb	Cell Signalling, 2125	1:1000
β-Actin	Ms	Sigma, A1978	1:2000
Goat anti-mouse IgG HRP	Gt	Abcam, ab6789	1:10,000
Goat anti-rabbit IgG HRP	Gt	Abcam, ab6721	1:10,000
Goat anti-rabbit IgG AF488	Gt	Life Technologies, A11008	1:200
Goat anti-chicken IgY AF594	Gt	Life Technologies, A11042	1:200
Goat anti-rat IgG AF488	Gt	Life Technologies, A11006	1:200

### Immunohistochemistry

A previously established protocol from our lab was used (Benson et al., 2015). Briefly, fixed tissue was immersed in 4% PFA in 0.1 M PB overnight at 4°C and then transferred into 30% sucrose in 0.1 M PB for two nights at 4°C. Tissue was embedded in optimum cutting temperature compound (TissueTek OCT, Sakura Finetek, 4583). DRGs were cryosectioned (Leica CM1950) at –20°C with a thickness of 10 μm on glass slides. Tissue sections were blocked in 10% normal goat serum for 1 h and incubated in primary antibody overnight. Slides were washed in PBS-Tween (0.5% Tween 20 in 1× PBS) and incubated in secondary antibody for 45 min. Slides were counterstained with Vectashield mounting medium with DAPI (Vector Laboratories, H-1200). Using a Zeiss Axiocam MRm camera and Zeiss Observer Z1 inverted fluorescence microscope, 20× fluorescent images were obtained for analysis. Representative confocal images (63×) were acquired using Leica CTR6000 and PerkinElmer UltraView Vox confocal imaging system.

### Dissociated DRG cultures for electrophysiology

Immediately after extraction, lumbar DRGs (L3-L6) from CFA (*n* = 5), onset (*n* = 3), and chronic (*n* = 5) mice were immersed in ice-cold dissection solution (118 mM NaCl, 2.5 mM KCl, 1.3 μM MgSO_4_, 1.2 mM NaH_2_PO_4_, 5 mM MgCl_2_6H_2_O, 25 mM D-glucose, 26 mM NaHCO_3_, and 1.5 mM CaCl_2_). Shortly thereafter, DRGs were digested with 0.5-mg/ml trypsin (Sigma, catalogue number T-9201), 1-mg/ml collagenase Type IV (Cedarlane, catalogue number LS004186), and 0.1-mg/ml deoxyribonuclease I (Sigma, catalogue number D-5025) dissolved in DMEM supplemented with GlutaMax (Invitrogen, catalogue number 10569044) for 40 min in a shaking water bath set at 35°C. Dissociated cells were plated onto 35 × 10-mm plates (VWR, catalogue number CA25382331) that were pretreated with 3-μg/ml poly-DL-ornithine (Sigma, catalogue number P-8638) dissolved in HPLC water (Sigma) and 2-μg/ml laminin (Sigma, catalogue number L-2020) dissolved in HBSS (138 mM NaCl, 5.33 mM KCl, 0.44 mM KH_2_PO_4_, 0.5 mM MgCl_2_ 6H_2_O, 0.41 mM MgSO_4_ 7H_2_O, 4 mM NaHCO_3_, 0.3 mM Na_2_HPO_4_, 5.6 mM D-glucose, and 1.26 mM CaCl_2_). Each 35 × 10-mm dish was immersed in 2-ml of culture medium (20 ml total), which contained 18-ml DMEM+GlutaMax (Invitrogen, catalogue number 10569044), 2-ml heat-inactivated horse serum (Sigma, catalogue number H-1138), 200-μl antibiotic-antimycotic 100× (Invitrogen, catalogue number 15240-096), and 20-μl antimitotic [cytosine β-D-arabinofuranoside (Ara-C), uridine, 5-fluoro-2’-deoxyuridine all at 10 μM (Sigma, catalogue numbers C1768, U3003, and F0503)]. Finally, cells were incubated at 36.5°C, 95% air–5% CO_2_ for 2–6 h.

### Electrophysiological recording

As described previously (Abdulla and Smith, 2001b), whole-cell patch-clamp experiments were done at room temperature (22°C) in bridge balance current-clamp mode using an NPI (model SEC 05 LX) amplifier (NPI Electronic GmbH). Whole-cell recording was established using a glass patch electrode (4–6 MΩ) containing internal solution comprised of 130 mM K gluconate, 4 mM Mg-ATP, 0.3 mM Na-GTP, 10 mM EGTA, 2 mM CaCl_2_, and 10 mM HEPES (adjusted to pH 7.2 with KOH; osmolarity 310–320 mOsm). The Petri dish was superfused with external solution containing 127 mM NaCl, 2.5 mM KCl, 1.2 mM NaH_2_PO_4_, 26 mM NaHCO_3_, 2.5 mM CaCl_2_, 1.3 mM MgSO_4_, and 25 mM D-glucose saturated with 95% O_2_–5% CO_2_ at ∼2 ml/min. All cells during current-clamp experiments were held at –60 mV. Neurons with resting membrane potential (RMP) less negative than –40 mV were rejected. DRG neuron excitability was assessed by counting total number of APs discharged in response to a 450-ms depolarizing current ramp to +2 nA. Rheobase was determined by measuring the amplitude of a 5 ms square wave depolarizing current pulse that was required to generate a single AP. Other spike parameters (Table 3) were measured as previously described (Stemkowski and Smith, 2012; Stemkowski et al., 2015). Data were obtained and analyzed using pCLAMP 10 (Molecular Devices).

**Table 3.** Various spike parameters of small (<26 µm) and large (≥26 µm) diameter DRG neurons obtained from CFA, EAE onset, and EAE chronic mice

	<26 µm			≥26 µm		
Spike parameter	CFA	Onset	Chronic	CFA	Onset	Chronic
Peak amplitude (mV)	116.4 ± 2.159	112.5 ± 2.437	116.2 ± 2.42	104.9 ± 1.796	102.2 ± 2.066	108.9 ± 1.362^#^
Afterhyperpolarization Amplitude (mV)	–14.38 ± 0.927	–10.22 ± 1.223^**^	–13.65 ± 0.525^#^	–13.84 ± 0.598	–12.18 ± 0.657	–12.38 ± 0.483
Half width (ms)^&^	3.959 ± 0.421	5.165 ± 0.915	4.213 ± 0.348	1.18 ± 0.104	1.474 ± 0.141	1.233 ± 0.083
Rise slope (mV/ms)	175.3 ± 22.55	83.64 ± 6.219^**^	143.1 ± 10.36^##^	236.7 ± 14.12	108.5 ± 4.298^***^	218.8 ± 12.5^###^
Decay slope (mV/ms)	–62.46 ± 6.376	–45.32 ± 8.131	–55.19 ± 5.451	–134.4 ± 5.835	–96.5 ± 5.518^***^	–120.4 ± 4.876^#^
Rheobase (nA)^&^	0.2625 ± 0.017	0.3 ± 0.023	0.3074 ± 0.018	0.4697 ± 0.028	0.349 ± 0.027^**^	0.4138 ± 0.022

Mean ± SEM. One-way ANOVA followed by Tukey’s test performed within each group; *p < 0.05, **p < 0.01, ***p < 0.001, in comparison to CFA; #*p* < 0.05, ##*p* < 0.01, ###*p* < 0.001 in comparison to Onset. ^&^Graphed in Figure 6.

### Experimental design and statistical analysis

Mice were randomly assigned to each experimental group. All statistical analyses were performed using GraphPad Prism 6. Data were subjected to one-way ANOVAs followed by Tukey’s test for pairwise comparisons. A Student’s *t* test was used when comparing only two groups. PCR and Western blotting data were log2 transformed and then analyzed to fulfill the normality and homogeneity of variance assumption for ANOVAs. Log-transformed data are back-transformed on a linear scale and graphed accordingly for the ease of the reader; α = 0.05 was used throughout the study. Statistical analyses are summarized in Table 4.

**Table 4.** Statistical analyses performed in this study

Figure	Data structure	Statistical test	Sample size	Statistical data
1	Log2-transformed to normalize data	One-way ANOVA (Tukey’s *post hoc* test)	CFA: 5 Onset: 5 Chronic: 5	A: *F*_(2,12)_ = 17.78, *p* = 0.0003 B: *F*_(2,12)_ = 4.217, *p* = 0.0410 C: *F*_(2,12)_ = 8.126, *p* = 0.0059 Di: *F*_(2,12)_ = 34.37, *p* < 0.0001 Dii: *F*_(2,12)_ = 42.74, *p* < 0.0001 Diii: *F*_(2,12)_ = 42.83, *p* < 0.0001 Div: *F*_(2,12)_ = 9.410, *p* = 0.0035
2*A*	Normal	One-way ANOVA (Tukey’s *post hoc* test)	CFA: 4 Onset: 5 Chronic: 5	*F*_(2,11)_ = 17.51, *p* = 0.0004
2*B*	Normal	One-way ANOVA (Tukey’s *post hoc* test)	CFA: 5 Onset: 5 Chronic: 3	*F*_(2,10)_ = 6.254, *p* = 0.0173
2*C*	Normal	One-way ANOVA (Tukey’s *post hoc* test)	CFA: 4 Onset: 5 Chronic: 4	*F*_(2,10)_ = 6.361, *p* = 0.0165
3*A–C*	Log2-transformed to normalize data	One-way ANOVA (Tukey’s *post hoc* test)	CFA: 4 Onset: 3 Chronic: 3	A: *F*_(2,7)_ = 0.9470, *p* = 0.4325 B: *F*_(2,7)_ = 2.747, *p* = 0.1317 C: *F*_(2,7)_ = 0.9616, *p* = 0.4276
3*D–I*	Log2-transformed to normalize data	One-way ANOVA (Tukey’s *post hoc* test)	CFA: 5 Onset: 5 Chronic: 5	D: *F*_(2,12)_ = 0.7474, *p* = 0.4944 E: *F*_(2,12)_ = 1.770, *p* = 0.2120 F: *F*_(2,12)_ = 0.8078, *p* = 0.4687 G: *F*_(2,12)_ = 10.52, *p* = 0.0023 H: *F*_(2,12)_ = 9.242, *p* = 0.0037 I: *F*_(2,12)_ = 12.56, *p* = 0.0011
4*C*	Non-normal	Two-tailed unpaired *t* test with Welch’s correction	CFA: 5 EAE: 13	*t*_(12.41)_ = 3.237, *p* = 0.0069
5*A–E*	Log2-transformed to normalize data	One-way ANOVA (Tukey’s *post hoc* test)	CFA: 5 Onset: 4 Chronic: 4	A: *F*_(2,10)_ = 10.73, *p* = 0.0032 B: *F*_(2,10)_ = 29.40, *p* < 0.0001 C: *F*_(2,10)_ = 4.361, *p* = 0.0435 D: *F*_(2,10)_ = 25.80, *p* = 0.0001 E: *F*_(2,10)_ = 15.92, *p* = 0.0011
5*H*	Normal	One-way ANOVA (Tukey’s *post hoc* test)	CFA: 4 Onset: 6 Chronic: 6	*F*_(2,13)_ = 7.750, *p* = 0.0061
6*C*	Non-parametric	Kruskal–Wallis *H* test	<26 µm: CFA: 33 Onset: 17 Chronic: 27 ≥26 µm: CFA: 76 Onset: 51 Chronic: 94	<26 µm: *H*_0.5nA(2)_ = 0.3138, *p* = 0.8548 *H*_1.0nA(2)_ = 0.1677, *p* = 0.9196 *H*_1.5nA(2)_ = 0.8715, *p* = 0.2750 *H*_2.0nA(2)_ = 0.9634, *p* = 0.6177 ≥26 µm: *H*_0.5nA(2)_ = 1.498, *p* = 0.4728 *H*_1.0nA(2)_ = 3.924, *p* = 0.1406 *H*_1.5nA(2)_ = 7.448, *p* = 0.0241 *H*_2.0nA(2)_ = 9.943, *p* = 0.0069
6*D*	Normal	One-way ANOVA (Tukey’s *post hoc* test)	<26 µm: CFA: 24 Onset: 13 Chronic: 27 ≥26 µm: CFA: 76 Onset: 51 Chronic: 94	*F*_<26µm(2,61)_ = 1.849, *p* = 0.1660 *F*_≥26µm(2,219)_ = 5.274, *p* = 0.0058
6*G*	Normal	One-way ANOVA (Tukey’s *post hoc* test)	CFA: 24 Onset: 13 Chronic: 27	*F*_(2,61)_ = 1.238, *p* = 0.2971
6*H*	Normal	Two-way ANOVA (Tukey’s *post hoc* test)	CFA: 31 Onset: 17 Chronic: 25	Disease: *F*_(2,444)_ = 2.740, *p* = 0.0657 Spike number: *F*_(7,444)_ = 25.00, *p* < 0.0001 Interaction: *F*_(14,444)_ = 0.1811, *p* = 0.9996
6*J*	Normal	One-way ANOVA (Tukey’s *post hoc* test)	CFA: 76 Onset: 52 Chronic: 94	*F*_(2,219)_ = 1.832, *p* = 0.1625
6*K*	Normal	Two-way ANOVA (Tukey’s *post hoc* test)	CFA: 40 Onset: 27 Chronic: 44	Disease: *F*_(2,708)_ = 38.03, *p* < 0.0001 Spike number: *F*_(7,708)_ = 11.82, *p* < 0.0001 Interaction: *F*_(14,708)_ = 0.6171, *p* = 0.8522

Table 3	Normal and non-normal	One-way ANOVA (Tukey’s *post hoc* test) or Kruskal–Wallis *H* test	<26 µm: CFA: 24 Onset: 13 Chronic: 27 ≥26 µm: CFA: 76 Onset: 51 Chronic: 94	Peak amplitude: *F*_<26µm(2,61)_ = 0.6022, *p* = 0.5508 *F*_≥26µm(2,219)_ = 3.883, *p* = 0.0220 Afterhyperpolarization amplitude: *F*_<26µm(2,61)_ = 5.211, *p* = 0.0081 *F*_≥26µm(2,219)_ = 2.481, *p* = 0.0860 Half width (as plotted in Fig. 6): *F*_<26µm(2,61)_ = 1.238, *p* = 0.2971 *F*_≥26µm(2,219)_ = 1.832, *p* = 0.1625 Rise slope: *H*_<26µm(2)_ = 14.44, *p* = 0.0007 *H*_≥26µm(2)_ = 50.99, *p* < 0.0001 Decay slope: *F*_<26µm(2,61)_ = 1.423, *p* = 0.2488 *F*_≥26µm(2,219)_ = 10.08, *p* < 0.0001 Rheobase (as plotted in Fig. 6): *F*_<26µm(2,61)_ = 1.849, *p* = 0.1660 *F*_≥26µm(2,219)_ = 5.274, *p* = 0.0058

## Results

### Immune activation and inflammation in the DRG of MOG-EAE mice

**Figure 1.**
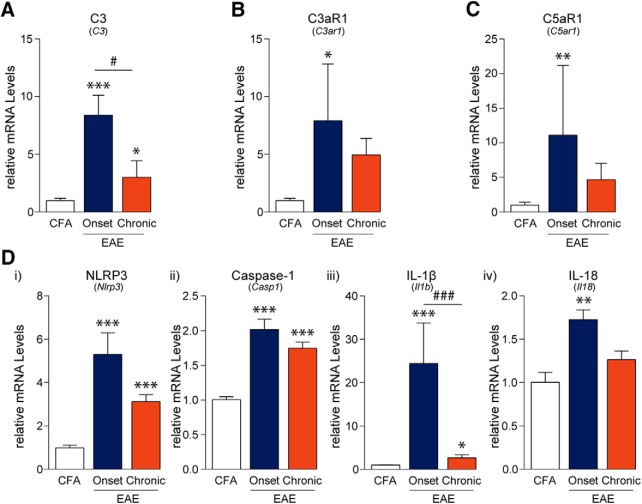
EAE-induced complement and inflammasome activation in the DRG. ***A–C***, PCR analysis of lumbar DRGs from EAE animals revealed that the complement component 3 (C3), its receptor C3aR1, and component 5a receptor (C5aR1; also known as CD88) are transiently upregulated at the onset of disease as compared to CFA control samples. ***D***, Similarly, mRNA transcripts of NLRP3, caspase-1, IL-1β, and IL-18 are also upregulated at disease onset only to taper off at the chronic time point 21 d post-induction. NLRP3 = NACHT, LRR, and PYD domains-containing protein 3; IL = interleukin. Bars indicate mean ± SEM; *,#*p* < 0.05; **,##*p* < 0.01; ***,###*p* < 0.001, one-way ANOVAs with Tukey’s *post hoc* analysis. CFA, *n* = 5; onset, *n* = 5; chronic, *n* = 5.

Since the activation of the immune system is an integral part of EAE pathogenesis, we examined lumbar DRGs for indications of immune activation and inflammation. Innate immune responses including complement system activation and subsequent initiation of the NLRP3 inflammasome are important mediators that activate and recruit adaptive immune cells (Hemmer et al., 2015; Arbore et al., 2016). Using PCR, we assessed the mRNA levels of complement components and receptors (C3, C3aR1, C5aR1) each of which were significantly altered throughout the disease course (*F*_C3(2,12)_ = 17.78, *p* = 0.0003; *F*_C3aR1(2,12)_ = 4.217, *p* = 0.0410; *F*_C5aR1(2,12)_ = 8.126, *p* = 0.0059, one-way ANOVA; Fig. 1*A–C*). *Post hoc* analysis using the Tukey’s multiple comparisons test indicated that C3, C3aR1, and C5aR1 mRNA expression was increased at the onset of disease in the DRG as compared to CFA controls. In addition, C3 mRNA expression was significantly elevated in samples from chronic time points as compared to CFA controls; however, these levels were significantly lower when compared to samples from the “onset” time point. Furthermore, the mRNA expression of the NLRP3 inflammasome components, NLRP3, caspase-1, and the inflammatory cytokines, IL-1β and IL-18, were significantly upregulated over the entire disease course (*F*_NLRP3(2,12)_ = 34.37, *p* < 0.0001; *F*_Caspase-1(2,12)_ = 42.74, *p* < 0.0001; *F*_IL-1β(2,12)_ = 42.83, *p* < 0.0001; *F*_IL-18(2,12)_ = 9.410, *p* = 0.0035, one-way ANOVA; Fig. 1*D*). NLRP3, caspase-1, and IL-1β transcript levels were increased at MOG-EAE onset and, to a lesser extent, at the chronic phase of MOG-EAE as compared to CFA samples. On the other hand, IL-18 transcript levels increased only at disease onset and then normalized back to non-diseased, CFA levels at the chronic time point.

**Figure 2.**
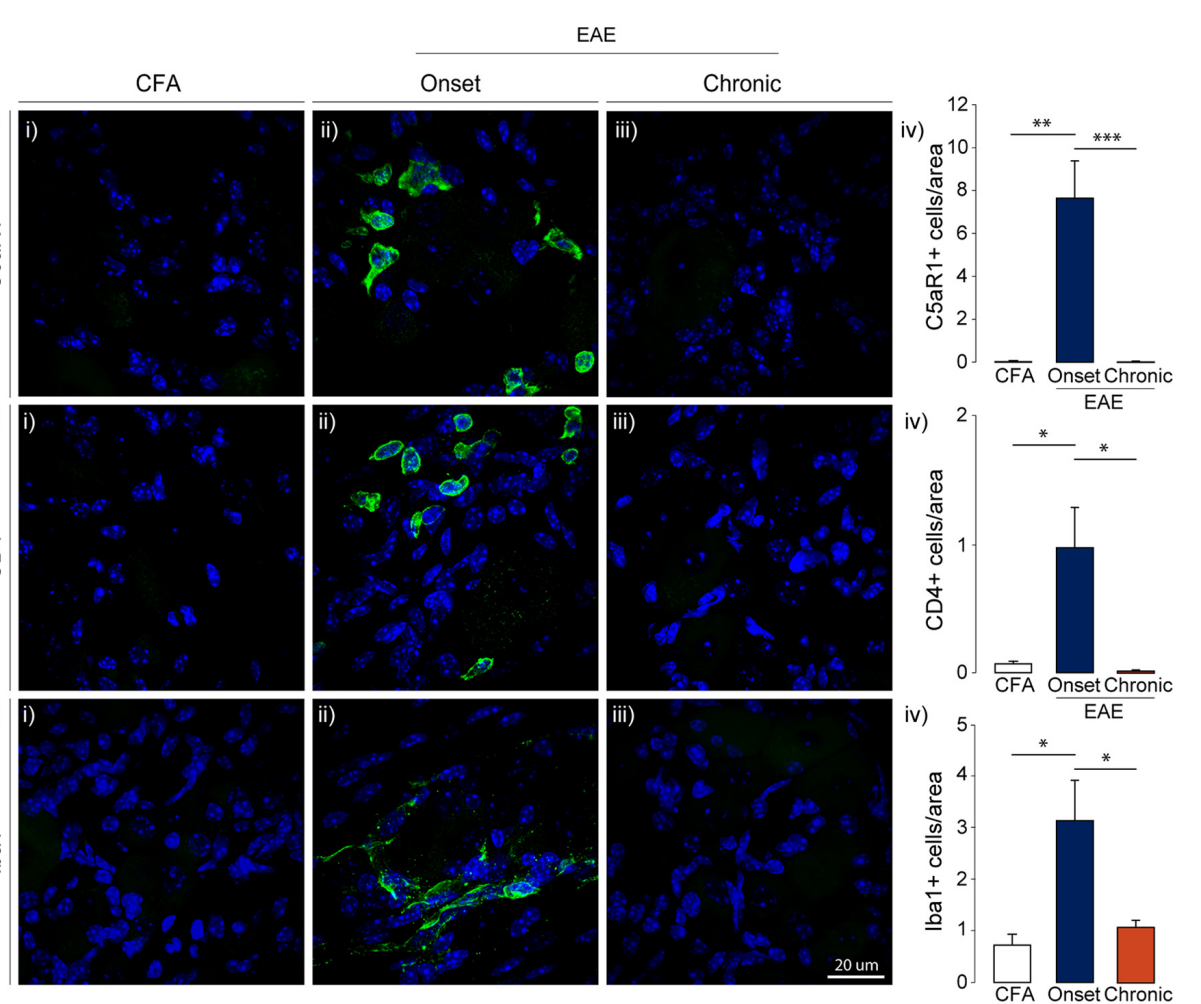
Immune cells infiltrate the DRG in EAE. ***A–C***, IHC analysis further confirmed that C5aR1+ immune cells, CD4+ T-cells, and Iba1+ macrophages infiltrate the DRG at EAE onset and retreat at the later, chronic disease stage. C5aR1 = complement 5a receptor; CD4 = cluster of differentiation 4; Iba1 = ionized calcium binding adapter molecule 1. Bars indicate mean ± SEM; *p < 0.05, **p < 0.01, ***p < 0.001, one-way ANOVAs with Tukey’s *post hoc* analysis. CFA, *n* = 5; onset, *n* = 5; chronic, *n* = 5.

Initial IHC analysis of C5aR1 revealed that this receptor, based on morphology, was transiently present in non-neuronal cells in the DRG at disease onset (*F*_C5aR1(2,11)_ = 17.51, *p* = 0.0004, one-way ANOVA; Liang et al., 2012). This is consistent with reports suggesting that macrophages are the primary source of C5aR1 in the DRG (Shutov et al., 2016). Further analysis of CD4+ T-cells and Iba1+ macrophages also showed a transient expression of infiltrating immune cells in the lumbar DRGs from mice with MOG-EAE (*F*_CD4(2,10)_ = 6.254, *p* = 0.0173; *F*_Iba1(2,10)_ = 6.361, *p* = 0.0165, one-way ANOVA; Fig. 2*A–C*). The number of these cells increased dramatically in the DRG at disease onset only to return to non-disease, CFA control levels at the chronic time point. Taken together, these results demonstrate that immune infiltration in the DRG of mice with MOG-EAE is a transient phenomenon that accompanies the onset of clinical signs of the disease.

### Myelin dysregulation

**Figure 3.**
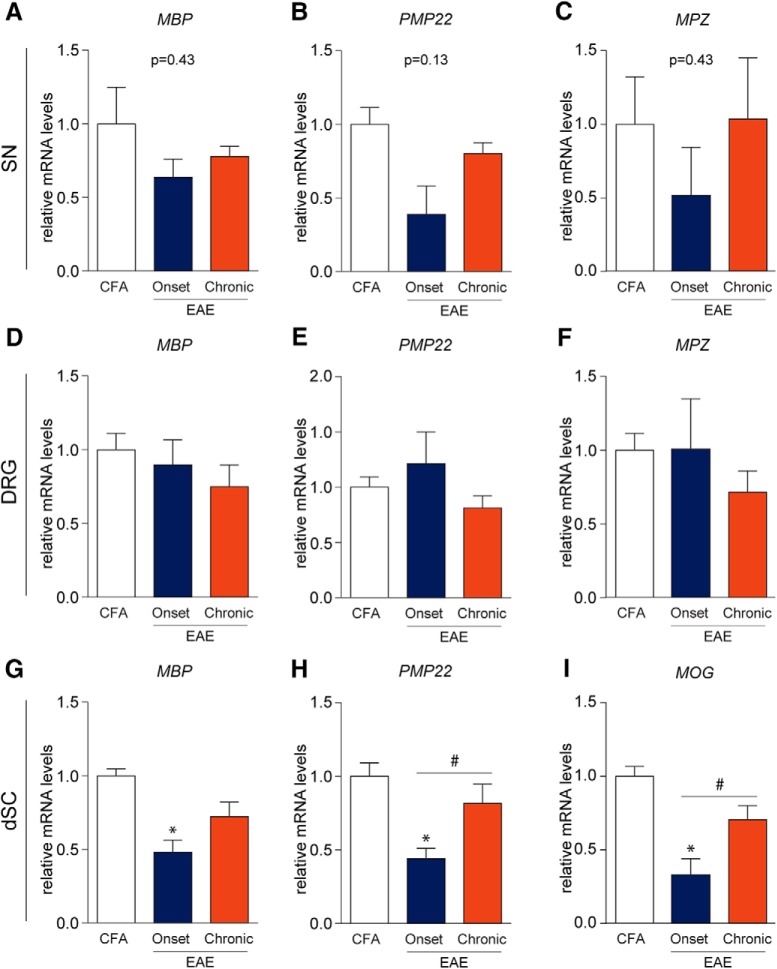
Myelin protein transcripts in the periphery are not significantly altered in EAE. ***A–F***, mRNA transcripts of MBP, PMP22, and MPZ were not significantly altered in EAE. ***G–I***, MBP, PMP22, and MOG transcripts were reduced at EAE onset in the dSC suggesting myelinopathy. Normalization of these transcripts was also observed chronically which may indicate repair mechanisms; *,#*p* < 0.05, one-way ANOVAs with Tukey’s *post hoc* analysis. ***A–C***, CFA, *n* = 4; onset, *n* = 3; chronic, *n* = 3. ***D–I***, CFA, *n* = 5; onset, *n* = 5; chronic, *n* = 5.

Since demyelination is another hallmark feature of MS, we investigated myelin disruption in the sciatic nerve (SN), DRG, and dorsal horn of the spinal cord (dSC) by assessing mRNA transcripts of important myelin structural proteins. In the SN and DRG, mRNA transcripts of myelin basic protein (MBP), peripheral myelin protein 22 (PMP22), and myelin protein zero (MPZ) were not significantly altered with the progression of MOG-EAE (SN: *F*_MBP(2,7)_ = 0.9470, *p* = 0.4325; *F*_PMP22(2,7)_ = 2.747, *p* = 0.1317; *F*_MPZ(2,7)_ = 0.9616, *p* = 0.4276; DRG: *F*_MBP(2,12)_ = 0.7474, *p* = 0.4944; *F*_PMP22(2,12)_ = 1.770, *p* = 0.2120; *F*_MPZ(2,12)_ = 0.8078, *p* = 0.4687, one-way ANOVA; Fig. 3). Since MOG expression in the PNS has recently been implicated in MOG-EAE pathology (Wang et al., 2017), we also performed qRT-PCRs for MOG transcripts in the DRG and the SN (data not shown) but were unable to reliably detect MOG transcripts, especially in the SN. In contrast, there was a significant downregulation of MBP, PMP22, and MOG mRNA in the dorsal horn at disease onset (*F*_MBP(2,12)_ = 10.52, *p* = 0.0023; *F*_PMP22(2,12)_ = 9.242, *p* = 0.0037; *F*_MOG(2,12)_ = 12.56, *p* = 0.0011, one-way ANOVA; Fig. 3). These transcript levels rebound at the chronic stage. Taken together, these results establish that disruption of myelin occurs in the dorsal horn while not being significantly impacted in the PNS over the disease course.

### Cellular injury and cytoskeletal disruption

**Figure 4.**
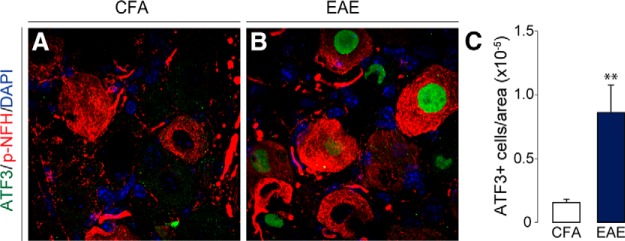
Cellular injury marker, ATF3, is upregulated in the DRG of EAE animals. ***A–C***, ATF3 expression in the nucleus of DRG neurons is induced with the onset of disease signs. P-NFH staining is used to identify neurons. ***C***, The number of ATF3-positive neurons in the DRG were normalized to the area (in pixels) of the entire DRG. Bars indicate mean ± SEM; **p < 0.01, two-tailed unpaired *t* test with Welch’s correction. CFA, *n* = 5; EAE, *n* = 13.

**Figure 5.**
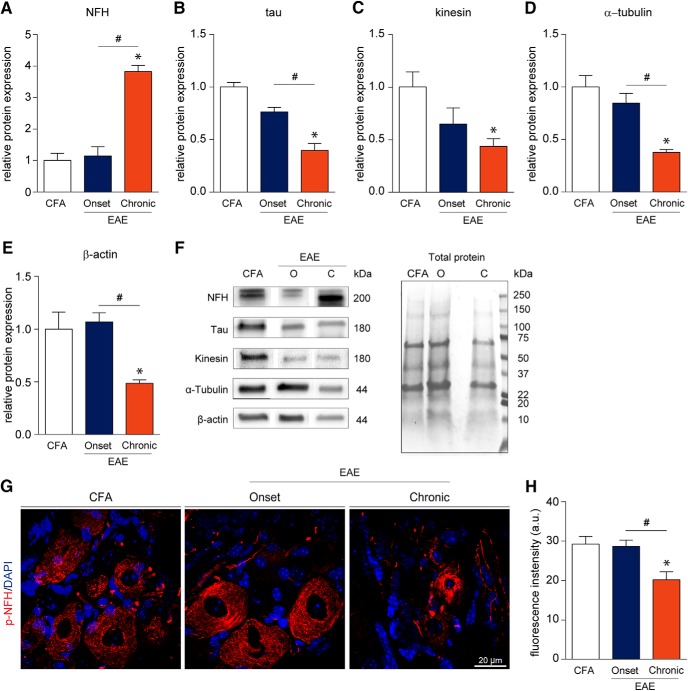
Cytoskeletal disruption of DRG neurons occurs late in the EAE disease course. ***A–F***, Western blotting data suggest that cytoskeletal proteins remain intact at the onset of EAE symptoms and become impaired at the chronic time point. We observe a significant elevation in the level of the non-phosphorylated isoform of heavy-chain NFH at the chronic stage. On the contrary, a significant reduction in the levels of tau, kinesin, α-tubulin, and β-actin was detected chronically. ***G***, ***H***, Immunofluorescence staining of lumbar DRGs for the p-NFH revealed a significant reduction in fluorescence intensity in chronic samples (20.23 ± 1.976 a.u.) as compared to CFA control (29.26 ± 1.951 a.u.) and EAE onset samples (28.68 ± 1.581 a.u.). Furthermore, p-NFH staining in the soma of chronic DRG neurons displays aberrant morphology. a.u. = arbitrary units. Bars indicate mean ± SEM. O = onset; C = chronic; *,#*p* < 0.05, one-way ANOVAs with Tukey’s *post hoc* analysis. ***A–F***, CFA, *n* = 5; onset, *n* = 4; chronic, *n* = 4. ***G***, ***H***, CFA, *n* = 4; onset, *n* = 6; chronic, *n* = 6.

Activating transcription factor 3 (ATF3) is a commonly used marker for assessing cellular injury in neurons (Frezel et al., 2016). In our study, we observed a marked upregulation of ATF3 in the DRG of MOG-EAE animals at the onset of disease signs (*t*_(12.41)_ = 3.237, *p* = 0.0069, Two-tailed unpaired *t* test with Welch’s correction; Fig 4). A common feature of MS is axonal damage and subsequent neurodegeneration in response to demyelination in the CNS (Haines et al., 2011). To assess whether cellular architecture was also affected in the PNS of EAE animals, we examined the levels of select cytoskeletal proteins [non-phosphorylated neurofilament-H (NFH), tau, kinesin, α-tubulin, and β-actin] over the disease course (Fig. 5*A–F*). Although cytoskeletal proteins from the PNS remained intact at the onset of disease, chronic disease lead to significant dysregulation of these cytoskeletal proteins (*F*_NFH(2,10)_ = 10.73, *p* = 0.0032; *F*_tau(2,10)_ = 29.40, *p* < 0.0001; *F*_kinesin(2,10)_ = 4.361, *p* = 0.0435; *F*_α-tubulin(2,10)_ = 25.80, *p* = 0.0001; *F*_β-actin(2,9)_ = 15.92, *p* = 0.0011, one-way ANOVA). Of note, there was a significant decrease in tau, kinesin, α-tubulin, and β-actin as compared to CFA controls. In contrast, NFH was increased dramatically at the chronic time point implicating axonal damage at this stage of disease progression (Fig. 5*A*). Further IHC analysis revealed that the average fluorescence intensity of phosphorylated NFH (p-NFH, NF200) was only reduced in the DRGs of chronically diseased animals (*F*_p-NFH(2,13)_ = 7.750, *p* = 0.0061, one-way ANOVA; Fig. 5*G*,*H*). On visual inspection, p-NFH in the soma of chronic DRG neurons displayed irregular morphology with increased compaction and reduced fascicular staining indicating cytoskeletal disruption at this later time point in the disease.

### Larger diameter (≥26 µm) neurons are hyperexcitable in MOG-EAE

**Figure 6.**
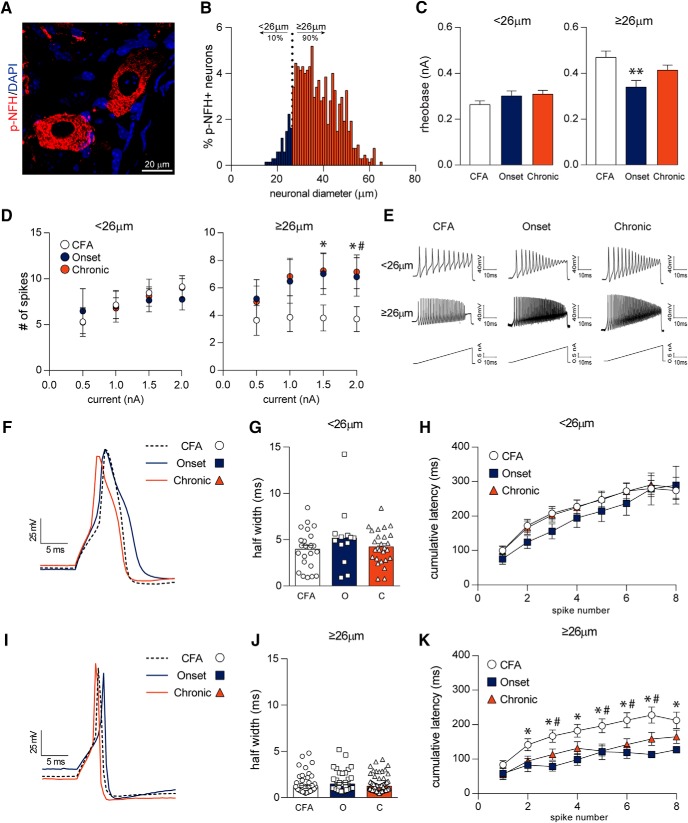
Larger diameter (≥26 µm) dissociated DRG neurons exhibit hyperexcitability. ***A***, ***B***, Labeling DRG sections from non-diseased control mice with p-NFH (also known as NF200) demonstrates that 90% of p-NFH+ cells are ≥26 µm, delineating smaller and larger cells. ***C***, ***D***, Dissociated DRG neurons from these animals exhibit aberrant firing properties under current ramp analysis. In particular, larger diameter (≥26 µm) EAE sensory neurons at disease onset have reduced rheobase and fire more APs with a current ramp of 1.5 and 2.0 nA than their CFA counterparts. EAE chronic DRG neurons also exhibit increased firing pattern at a current ramp of 2.0 nA but show an insignificant reduction in their rheobase. ***E***, Sample 2.0-nA current ramp traces of dissociated DRG neurons. ***F–H***, Half width of smaller diameter neurons (<26 µm) as well as cumulative latencies of APs remain relatively unchanged with EAE disease. ***I–K***, Although spike width is unaffected in larger diameter neurons (≥26 µm), cumulative latencies are reduced with EAE disease indicating that APs fire quicker in succession with the onset of EAE. Other spike parameters are summarized in Table 3. ***C***, *,#*p* < 0.05, Kruskal–Wallis *H* test. * CFA versus Onset; # CFA versus Chronic. ***D***, **p < 0.01, one-way ANOVAs with Tukey’s *post hoc* analysis. ***K***, *,#*p* < 0.05, two-way ANOVA with Tukey’s *post hoc* analysis. ***A***, ***B***, *n* = 5, 674 of 1055 cells were p-NFH+. ***C***, ***D***, <26 µm: CFA, *n* = 33; onset, *n* = 17; chronic, *n* = 27; ≥26 µm: CFA, *n* = 76; onset, *n* = 51; chronic, *n* = 94. ***G***, CFA, *n* = 24; onset, *n* = 13; chronic, *n* = 27. ***H***, CFA, *n* = 31; onset, *n* = 17; chronic, *n* = 25. ***J***, CFA, *n* = 76; onset, *n* = 52; chronic, *n* = 94. ***K***, CFA, *n* = 40; onset, *n* = 27; chronic, *n* = 44.

To assess the functional consequence of the disease in the DRG of EAE mice, we next conducted an electrophysiological assessment of the sensory neurons. p-NFH (also known as neurofilament 200) has previously been used to identify larger-diameter, putative myelinating cells (Ruscheweyh et al., 2007; Usoskin et al., 2015; Xu et al., 2015). Labeling of control, non-EAE DRG tissue with p-NFH revealed a spectrum of DRG neurons, 90% of which were greater than or equal to 26 µm in diameter, making 26 µm a good benchmark for delineating smaller and larger cells (Fig. 6*A*,*B*). The minimal amount of current required to elicit an AP, also known as rheobase, of smaller diameter neurons (<26 µm) remained unchanged while larger diameter neurons (≥26 µm) exhibited a reduced rheobase with the onset of MOG-EAE as compared to CFA cells (*F*_<26µm(2,61)_ = 1.849, *p* = 0.1660, *F*_≥26µm(2,219)_ = 5.274, *p* = 0.0058, one-way ANOVA; Fig. 6*C*). Smaller diameter (<26 µm) dissociated neurons from this cohort revealed no difference in number of APs on current ramps as compared to cells from non-diseased, CFA mice (Fig. 6*D*,*E*). In contrast, larger diameter neurons (≥26 µm) from EAE mice at the onset and chronic time points fire more APs in response to current ramps of 1.5 and 2.0 nA (*H*_1.5nA(2)_ = 7.448, *p* = 0.024; *H*_2.0nA(2)_ = 9.943, *p* = 0.007, Kruskal–Wallis *H* test; Fig. 6*D*,*E*). Deeper analysis of individual APs demonstrated transient changes in spike parameters in both smaller and larger diameter neurons in the disease (Fig. 6*F*,*I*; further summarized in Table 3). Spike width was unaltered in both size categories with the progression of disease (*F*_<26µm(2,61)_ = 1.238, *p* = 0.2971, *F*_≥26µm(2,219)_ = 1.832, *p* = 0.1625, one-way ANOVA; Fig 6*G*,*J*). Larger diameter DRG neurons from MOG-EAE animals, both at onset and chronic time points, also fire consecutive APs much quicker than CFA controls, as measured by their cumulative latencies (disease: *F*_≥26µm(2,708)_ = 38.03, *p* < 0.0001, spike number: *F*_≥26µm(7,708)_ = 11.82, *p* < 0.0001, interaction: *F*_≥26µm(14,708)_ = 0.6171, *p* = 0.8522, two-way ANOVA; Fig. 6*K*). These results indicate that DRG neurons ≥26 µm become hyperexcitable in MOG-induced EAE.

## Discussion

MS and its commonly used animal model, MOG-EAE, has traditionally been viewed as a disorder affecting the CNS. Here, we provide evidence in addition to that notion, suggesting that the DRG of mice with MOG-EAE also undergo various pathologic changes. Sensory neurons experience inflammation and cellular injury with progression of the disease. Electrophysiological analysis of neurons from the lumbar DRG of MOG-EAE mice also reveals a lasting functional consequence of MOG-EAE as demonstrated by an increased excitability of medium-to-large diameter neurons.

While the cause of MS is not known, it is accepted that the disorder involves the activation of the immune system (Dendrou and Fugger, 2017). Although the EAE-inducing antigen is predominantly expressed in the CNS (i.e., MOG_35-55_ peptide), pathologic features of the disease are also found in the PNS. IHC and molecular analyses of the sensory ganglia reveal an infiltration of immune cells and an increase in cytokines, chemokines, and neurotrophic factors with disease progression. Infiltrating T-cells and macrophages have been observed in the DRG (Duffy et al., 2016; Frezel et al., 2016) and the TG (Duffy et al., 2016; Frezel et al., 2016; Thorburn et al., 2016) with the onset of EAE. In particular, the trigeminal nerve and ganglia experience an influx of CD3+ T-cells early on in the presymptomatic phase of the disease (∼day 8) followed by an increase in CD4+ T-cells at disease onset and peak (∼day 16; Duffy et al., 2016; Frezel et al., 2016; Thorburn et al., 2016) . CD4+ T-cells remain elevated in the TG and the trigeminal root entry zone (TREZ) chronically (∼day 35; Thorburn et al., 2016). The DRG on the other hand does not experience immune cell infiltration to the same extent as the trigeminal system. Although CD4+ T-cells infiltrate the lumbar DRG at peak disease (∼day 16), these immune cells dissipate with the progression of the disease (Duffy et al., 2016; Wang et al., 2017). Very few CD3+ T-cells are found in the diseased DRG (Duffy et al., 2016). Consistent with previous accounts, this study demonstrates a transient increase in CD4+ T-cells in the DRG of MOG-EAE mice, peaking at the onset of disease. Literature on the monocyte composition of the sensory ganglia during EAE has been limited. A recent study showed an increase in Iba1+ macrophages in the TG at the onset of MOG-EAE signs (Thorburn et al., 2016). Similarly, here we report for the first time an increase in Iba1+ macrophages in the DRG at the onset of MOG-EAE signs, only to normalize by the chronic time point.

On infection, the innate immune system is often the first to respond to a foreign substance by recruiting immune cells to the site of injury, initiating the complement cascade, and by activating the adaptive immune system (Turvey and Broide, 2010; Hemmer et al., 2015). In this regard, activation of innate immune cells, such as resident macrophages, can lead to the production of complement components and NLRP3-mediated cytokines including IL-1β and IL-18 (Turvey and Broide, 2010; Hemmer et al., 2015; Mathern and Heeger, 2015). Complement components C3a and C5a are known as anaphylatoxins and, as such, cause a local inflammatory response by binding to their receptors C3aR1 and C5aR1 (Griffin et al., 2007; Liang et al., 2012; Mathern and Heeger, 2015). C3a, C5a, and IL-1β have also previously been linked to nociceptor sensitization (Ho et al., 2010; Liang et al., 2012; Stemkowski et al., 2015). Our results provide the first evidence of a prolonged activation of the NLRP3 inflammasome and a transient activation of the complement system in the DRG of MOG-EAE mice. Despite the reduced presence of immune cells in the DRG at the chronic time point, increased *NLRP3*, *Casp1*, and *Il1b* transcripts may be produced by resident cells including neurons and satellite glial cells.

Demyelination is a canonical feature of MS and EAE. Early studies that examined the coccygeal dorsal roots in a rat model of EAE demonstrated that tail paralysis progressively coincided with demyelination and conduction block of lightly myelinated afferents (Pender, 1986; Pender and Sears, 1986). As a result, these rats displayed hypoesthesia with reduced vocalization on noxious mechanical stimulation of the tail (Pender, 1986). Many CNS myelin proteins, including MBP and PLP, are also found in the PNS (Nave and Werner, 2014) and thus induction with whole spinal cord lysates or MBP may lead to peripheral myelin reactive T-cells. In mice, Wang et al. (2017) noted myelin decompaction and dissociation in the sciatic nerve of both MOG-induced EAE and in MOG-EAE that was generated without pertussis toxin (EAEnp). Using nested qRT-PCRs, these authors confirmed that MOG transcripts are found in the peripheral nerves, speculating that this is the target of immune attack in the periphery. However, since no myelin loss was observed in the peripheral nerve and protein expression of MOG in the PNS *in vivo* was undetected (Pagany et al., 2003; Nave and Werner, 2014), it remains to be determined whether MOG transcripts in the PNS are indeed transcribed into proteins that can be targeted by immune cells.

Neurodegeneration is another hallmark pathologic feature of MS. In this regard, we observed a reduction in various cytoskeletal-associated proteins including tau, kinesin, α-tubulin, and β-actin in PNS samples at the chronic time point of MOG-EAE. Of note, non-phosphorylated NFH was significantly elevated while the phosphorylated isoform of NFH was downregulated. NFHs are the most phosphorylated protein in the nervous system (Kirkcaldie and Collins, 2016). A complex balance of phosphorylation and dephosphorylation of NFH, in collaboration with microtubule associated proteins (e.g., tau), actin, tubulin, and motor proteins (e.g., kinesin), allows for efficient movement across the axon and supports the survival of the neuron. We also observed that p-NFH in chronic samples had an irregular morphology with increased fragmentation and a loss of regular, round lattice structures. This is characteristic of p-NFH in injured cells (Siedler et al., 2014; Kirkcaldie and Collins, 2016). ATF3, a stress-induced transcription factor, is present presymptomatically (Frezel et al., 2016) and upregulated at onset of motor signs (Fig. 5). It is interesting to note that ATF3 expression can be induced by only CFA and pertussis toxin inoculation without the MOG peptide while the peptide is required for T-cell infiltration into the DRG (Frezel et al., 2016). ATF3 expression in our MOG-EAE model was found to be dramatically increased as compared to CFA-controls although to a much lesser degree than models of peripheral nerve injury (Tsujino et al., 2000; Hunt et al., 2012). Furthermore, evidence suggests that secondary injury pathways such as Ca^2+^ dysregulation and mitochondrial dysfunction precede cytoskeletal disruption (Siedler et al., 2014). We did not observe neuronal apoptosis, as measured by cleaved caspase-3 immunoreactivity (data not shown), in our cohort. However, activation of the NLRP3 inflammasome is known to induce another form of cell death, pyroptosis, via caspase-1 rather than caspase-3 (Man et al., 2017). This hypothesis will need to be addressed in future studies.

The gate control theory of pain (Melzack and Wall, 1965) proposes that local interneurons of the spinal cord modulate pain perception by integrating nociceptive and innocuous stimulation from the primary afferents before relaying information to the higher-order structures of the brain. Injury or disease can lead to sensitization of peripheral and/or central neurons facilitating, augmenting, potentiating, and amplifying their response, ultimately contributing to abnormal and persistent sensory processing (Latremoliere and Woolf, 2009). Here, we describe the first account of aberrant electrophysiological responses of DRG neurons in MOG-induced EAE. Electrophysiological analysis has revealed an increase in the excitability of small-diameter putative nociceptive neurons in other models of neuropathic pain (Garrison et al., 2014; Ratté and Prescott, 2016; Moy et al., 2017; Mule et al., 2018). In contrast, we find that larger diameter neurons (≥26 µm) in the DRG of MOG-EAE mice fire more APs at higher current ramps, have a lower rheobase, and reduced cumulative latency, all indicative of hyperexcitablity. Previous electrophysiological studies in the PNS of EAE animals have focused largely at the chronic time point during which, as shown here, immune cell activation subsides and cytoskeletal disruption prevails (Pender and Sears, 1982, 1986; Lu et al., 2012). In fact, one of the first studies investigating the involvement of the DRG in EAE noted demyelination-induced nerve conduction block in rats and rabbits up to 7 d after disease onset. It should be noted however that the inoculum used by Pender and Sears (1982, 1986) was a whole guinea pig spinal cord. As discussed in their original paper, the inoculum may contribute to peripheral demyelination since some myelin antigens that are present in the CNS are also present in the periphery (Pender and Sears, 1982, 1986; Nave and Werner, 2014). Just as the purpose of this study was to highlight novel mechanisms of sensory dysfunction in MOG-EAE, by performing those initial electrophysiological studies on whole spinal cord induced EAE, Pender and Sears (1982, 1986) brought into question the peripheral component of EAE and hence, its relevance to MS. More recently, Lu et al. (2012) demonstrated increased excitability of large-diameter Aβ primary afferents using skin-nerve preparations in SJL-PLP_139-151_ EAE mice although at the chronic time point (35–45 d after induction). Along with our current study, Lu et al. (2012) identify a disease-mediated effect on large diameter mechanoreceptors rather than small diameter nociceptors. In this regard, we consistently observe mechanical hypersensitivity in MOG_35-55_-induced EAE which may require heightened input from mechanoreceptors, as previously suggested (Xu et al., 2015; Xie et al., 2016; Sun et al., 2017). Of note, we do not reliably observe heat hyperalgesia in our model (data not shown) further corroborating the lack of electrophysiological changes in smaller diameter neurons (<26 µm) which are known to possess heat-sensing TRPV1 channels. Aberrant firing properties of larger diameter afferents (≥26 µm) may therefore be a driver of central sensitization that can maintain chronic mechanical hypersensitivity (Baron et al., 2013).

Inflammatory mediators, such as IL-1β (Binshtok et al., 2008; Stemkowski et al., 2015; Alles and Smith, 2018) and TNFα (Czeschik et al., 2008; Leung and Cahill, 2010; Wang et al., 2018), that are induced in EAE (Melanson et al., 2009; Rodrigues et al., 2016) can modulate ion channel expression and/or activity and induce neuronal hyperexcitability. The electrophysiological changes remaining into the chronic phase of MOG-EAE suggest that transient immune cell infiltration and activation in the DRG inherently alters neuronal properties into the chronic phase of MOG-EAE. In this regard, long-term IL-1β exposure (5–6 d) to dissociated rat DRG neurons increases the excitability of medium-sized sensory neurons in a potassium-channel dependent manner (Stemkowski and Smith, 2012; Stemkowski et al., 2015). At the chronic timepoint in our model, slightly elevated levels of IL-1β (Fig. 1) in concert with altered ion channels may contribute to electrophysiological changes (Fig. 6). Various models of neuropathic pain have reported changes in sensory neuron excitability. After peripheral nerve injury, sensory neurons have been consistently reported to have a reduced rheobase and increased excitability (Abdulla and Smith, 2001a,b). However, the cell-specific changes in neurophysiological properties of sensory neurons typically vary with the model of study. Spinal nerve ligation in rats induces mechanical hyperalgesia and allodynia and this phenomenon coincides with hyperexcitability of medium and large diameter DRG neurons (Ma et al., 2003). In comparison, partial rhizotomy reduces mechanical threshold (hyperalgesia) but does not induce allodynia in rats. Unlike spinal nerve ligation, partial rhizotomy does not significantly alter sensory neuron electrophysiology, suggesting involvement of central mechanisms to modulation pain (Ma et al., 2003). Other axotomy models have noted increased AP amplitude and reduced rheobase in mainly small unmyelinated C-fibers (Zhang et al., 1997). Small-diameter neurons in our model have increased afterhyperpolarization amplitude (Table 3) which may allow these cells to fire multiple AP in response to increasing stimulus frequency, as previously suggested (Villière and McLachlan, 1996). Differences between our study and the literature may also be attributed to differences in animals, strains, and even sexes.

Sensory axons projecting into the CNS encounter an inflammatory milieu which may initiate retrograde stress responses causing an infiltration of leukocytes to mitigate cellular injury or damage. Myelinated axons of sensory neurons are especially susceptible to EAE inflammation due to the active demyelination in the CNS. We describe here the first account of aberrant electrophysiology of DRG neurons in MOG_35-55_-induced EAE. Electrophysiological analysis have revealed increased excitability of putative nociceptive neurons in various models of neuropathic pain (Garrison et al., 2014; Ratté and Prescott, 2016; Moy et al., 2017; Mule et al., 2018). Contrary to this, we find that medium-to-large diameter neurons in the DRG of MOG-EAE mice fire more APs at higher current ramps and have a lower rheobase, indicative of hyperexcitablity. Pharmacologically blocking medium to large diameter afferents from the DRG using a combination of flagellin and QX-314 alleviates mechanical allodynia but not heat hyperalgesia in multiple models of neuropathic pain (Xu et al., 2015). Our electrophysiology data support this notion and demonstrate that large diameter neurons are the most affected in the PNS of MOG-EAE mice.

## Synthesis

Reviewing Editor: Karen Davis, Krembil Research Institute and University of Toronto Decisions are customarily a result of the Reviewing Editor and the peer reviewers coming together and discussing their recommendations until a consensus is reached. When revisions are invited, a fact-based synthesis statement explaining their decision and outlining what is needed to prepare a revision will be listed below. The following reviewer(s) agreed to reveal their identity: Gila Moalem-Taylor, Marzia Malcangio.

Several mechanisms have been implicated in pain associated with EAE and MS. In this study, the authors advance the field by showing DRG neuronal hyperexcitability indicating peripheral sensitization in mice with EAE. Additionally, the authors demonstrate some inflammatory changes within the DRG of EAE mice, including activation of the complement system and the NLRP3 inflammasome, infiltration of T cells and macrophages, neuronal injury and cytoskeleton dysregulation, in the absence of peripheral demyelination. These peripheral mechanisms may contribute to neuropathic pain in EAE. The authors conclude that the onset of EAE is associated with peripheral sensitization and immune cell infiltration in the DRG. To the authors admittance, this is not a novel concept.

This is a well-written manuscript and important study. The results are sound demonstrating several inflammatory changes within the DRG of EAE mice, including activation of the complement system and the NLRP3 inflammasome, infiltration of T cells and macrophages, neuronal injury and cytoskeleton dysregulation, all in the absence of peripheral demyelination. Most importantly, the authors show DRG neuronal hyperexcitability suggesting peripheral sensitization. However, the lack of data on changes in the spinal cord make it difficult to assess the biological relevance of the peripheral changes. The following specific comments should be addressed in a revision:

Major Comments:

1. The relationships between the findings are not presented and whether the observed changes contribute to neuropathic pain in EAE is inconclusive. Although each of the presented results appears solid, the correlation between the different data sets lacking. The most novel aspect of this manuscript is the electrophysiological changes in the DRG neurons, however, how these changes are related to the inflammatory observations and whether they are correlated with the severity of disease, the pain phenotype, or other sensory dysfunction of these mice is not presented.

2. Figure 2. It is difficult to assess the significance of markers expression, for instance a comparison between CD4 and Iba1 staining appears to indicate that more T cells are in DRG than macrophages. What is the biological significance of this phenomenon? I suggest they include quantification of the same markers in the spinal cord, for comparison purposes.

3. Figure 4. Similarly, what is the biological significance of the ATF-3 staining in an average of 1 cell/area? How does this compare to models of nerve injury?

4. Figure 6, Discussion lines 380- 388 This interesting and important critical section needs revision as it would appear that the authors had not read the first paper from Pender and Sears published in Brain. That work, like the present work, was specifically done then to challenge orthodox views about the validity of the EAE model in relation to MS. In that case the possibility that the guinea pig spinal cord used as antigen (albeit freed from spinal roots and investing membranes) might well contain peripheral-based antigens was well recognised. However it is clear from reading the earlier paper that the work was specifically done as a challenge to orthodoxy because the histo-pathological paper published by Waxman and Adams to introduce EAE contained clear evidence of DRG, spinal root, and root entry demyelinating lesions. So the present work carries an important lesson,namely that a widely adopted and accepted MOG-EAE model of MS carries a peripheral component just as Pender and Sears had established for EAE in the last century! I expect the authors to revise this section accordingly.

5. My main criticism would be that the authors have not included any discussion as to whether or not the natural impulse traffic in such a broad range of afferents would actually excite repetitive activity; large diameter cell bodies are known to have a long unmyelinated axon between cell body and the T- branch and according to Ito transmission can occur through the branch point without the cell body being activated. In the same vein, do they suppose that the terminals are also more excitable.

Bearing in mind the historical importance and value of 'rheobase' I recommend the authors make statements about rheobase first and not after they describe their results using a ramp excitation.

Other Comments:

1. Abstract - several abbreviations are not defined, including ATF3 and PNS. In line 14, 'and' should be an.

2. Methods - the concentration of the CFA and injection sites should be specified.

3. Results - The EAE scoring of the mice, and whether disease severity was correlated with any of the inflammatory/cytoskeletal changes are missing.

4. Page 9, Fig. 2 should be Fig. 1.

5. Page 10, first paragraph - how do you know that C5aR1 is expressed on non-neuronal cells without double-labeling?

6. Page 10 - Myelin dysregulation - Since there are several reports of MOG expression in the peripheral nervous system and since MOG was used as the immunizing antigen, why wasn't MOG expression tested in the SN and DRG?

7. Page 11 -Fig. 4 - The image shows also staining for p-NFH, which is not mentioned.

8. The results of NFH versus p-NFH expression and its description is a bit confusing. Why wasn't p-NFH tested by Western blot, as the other proteins? I also suggest inserting a short sentence on the difference between them.

9. Page 12 - The description of the changes in Fig. 6F,I is unclear, and the image looks like the action potential is more affected at the chronic stage, while the text states changes are at the onset of EAE (line 283).

10. Discussion - Page 14 first paragraph - the sentence 'Here, we provide evidence to the contrary, suggesting that the DRG of mice with EAE also undergo various pathological changes'. I suggest rephrasing this sentence, it is not to the contrary, but rather in addition to changes in the CNS there are also changes in the periphery, some of which have already been reported.

11. Page 14, line 301, I suggest removing 'exclusively', since as the authors note MOG has been previously shown to be expressed in the PNS (Pagany 2003 and Wang 2017).

12. In the Discussion, I suggest that the authors expand on the relationshi between the inflammatory/cytoskeletal changes and electrophysiological changes at the onset versus the chronic phase of EAE.

13. Fig. 6 C, H, K - symbols are missing.

14. Other text matters:

- Title. The model is the MOG-EAE. should read; T-cell mediated MOG-EAE model of MS

- line7 replace EAE by MOG- EAE as this is the only model studied.

-‘ 8 replace 'the' by this.

- 28 MOG-EAE replace commonly by 'currently'

- The acronym MOG-EAE should be used throughout except for the most general points so that the comparison in the introduction,discussion and elsewhere with EAE, the older and original model, is clearer for new (young!) readers.

line 75 suggest; We find that at the onset of disease DRGs become inflamed with immune cell invasion followed by.......

- 78 Suggest delete putative and keep to the facts.' large diameter neurones

- 118 Western

- 119 Briefly

- 129/30 Membranes were then washed

- 166 incubated at 36.5

- 210 replace 'dampened' by 'lower'
